# Hypoxia-targeted gold nanorods for cancer photothermal therapy

**DOI:** 10.18632/oncotarget.25492

**Published:** 2018-05-29

**Authors:** Yuan Chen, Xiaomei Bian, Maureen Aliru, Amit A. Deorukhkar, Oscar Ekpenyong, Su Liang, Jyothy John, Jing Ma, Xiuqing Gao, Jon Schwartz, Pankaj Singh, Yuanqing Ye, Sunil Krishnan, Huan Xie

**Affiliations:** ^1^ Department of Pharmaceutical and Environmental Sciences, College of Pharmacy and Health Sciences, Texas Southern University, Houston, TX, USA; ^2^ Department of Radiation Oncology, Division of Radiation Oncology, The University of Texas M. D. Anderson Cancer Center, Houston, TX, USA; ^3^ Nanospectra Biosciences, Inc., Houston, Texas, USA; ^4^ Department of Epidemiology, Division of OVP, Cancer Prevention and Population Science, The University of Texas M. D. Anderson Cancer Center, Houston, TX, USA

**Keywords:** gold nanorods, hypoxia, carbonic anhydrase IX, photothermal therapy, hyperspectral imaging

## Abstract

Tumor hypoxia is a well-recognized driver of resistance to traditional cancer therapies such as chemotherapy and radiation therapy. We describe development of a new nanoconstruct composed of gold nanorods (GNRs) conjugated to carbonic anhydrase IX (CAIX) antibody that specifically binds to CAIX, a biomarker of hypoxia, to facilitate targeting tumor hypoxic areas for focused photothermal ablation. Physicochemical characterization studies confirmed the size, shape, monodispersity, surface charge, and serum stability of the GNRs. Enzyme-linked immunosorbent assays and cellular binding and uptake studies confirmed successful conjugation of antibody to the GNRs and specificity for CAIX. Near-infrared irradiation of CAIX-overexpressing cells treated with GNR/anti-CAIX resulted in significantly higher cell death than cells treated with control GNRs. *In vivo* biodistribution studies using hyperspectral imaging and inductively coupled plasma mass spectrometry confirmed intravenous administration results not only in greater accumulation of GNR/anti-CAIX in tumors than control GNRs but also greater penetration into hypoxic areas of tumors. Near-infrared ablation of these tumors showed no tumor regression in the sham-treated group, regression but recurrence in the non-targeted-GNR group, and complete tumor regression in the targeted-GNR group. GNR/anti-CAIX nanoconstructs show promise as hypoxia targeting and photothermal ablation agents for cancer treatment.

## INTRODUCTION

A major public health concern and a leading cause of death, cancer, has motivated scientists to explore adjunctive or alternative interventions to standard treatments, such as surgery, radiation therapy, and chemotherapy. Standard cancer treatments can be highly toxic to healthy tissues without differentiating malignant from normal cells, causing significant adverse effects in patients. Nanoparticle-based photothermal ablation therapy assisted by near-infrared (NIR) laser is an evolving area of interest with the potential to eliminate large tumors, reduce cancer resistance and prevent recurrence [1–3]. The basic principle behind photothermal ablation therapy is that heat generated from plasmon resonance of atoms on the surface of nanoparticles illuminated with NIR light can be used to destroy cancer cells. Strong optical absorption and high efficiency of photothermal conversion at the cancer site are critical to the success of this therapy.

An ideal metal nanostructure to be used in photothermal ablation therapy should have effective delivery, strong and tunable surface plasmon resonance absorption in the NIR, a low toxicity profile, and selectivity in targeting cancer cells. Noble metal nanoparticles in general, and gold and silver analogs in particular, have attracted extensive interest and led to the development of a wide variety of nanocarriers owing to their fulfillment of many of the above properties. Substantial effort has been made to develop gold nanoparticles of various shapes and with various surfaces; nanospheres, nanorods and nanoshells have been broadly applied in this type of therapy [1–3]. Among them, gold nanorods (GNRs) have attracted particular attention for their efficiency of photothermal activation and smaller size than gold nanoshells for NIR activation [3–5]. However, poor accumulation of these nanoparticles in solid tumors remains a significant challenge for photothermal ablation therapy [6].

Increasing attention has been focused on targeting specific pathways in the growth and development of tumors, especially the tumor microenvironment [7–12], as a way to improve the uptake of nanoparticles by tumors. Most tumors develop areas of hypoxia as proliferating cells outgrow the new vasculature that feeds them oxygen and nutrients, resulting in a gradient of increasing hypoxia that extends from the feeding vessel [13]. Tumor hypoxia usually occurs at a distance of 100∼200 μm from blood vessels and is a key factor predicting the response of tumors to irradiation; hypoxic cells are up to three times more resistant to radiation than normoxic cells because full radiosensitization requires the presence of endogenous oxygen that is ionized and fixes DNA damage [13, 14].

Hypoxia inducible factor 1 (HIF-1), a key transcription factor, is critically involved in the transcription of a spectrum of genes that regulate cellular survival under hypoxic conditions. Carbonic anhydrase IX (CAIX) is one such metalloenzyme, the expression of which is exclusively upregulated by activated HIF-1 [15]. CAIX is a member of the carbonic anhydrase family that catalyzes the rapid conversion of carbon dioxide to bicarbonate and protons. Presenting in few normal human tissues, the CAIX protein is highly expressed in hypoxic zones within human epithelial tumors derived from tissues that ordinarily do not express this isozyme [16]. The expression of CAIX is generally very low in some carcinoma cell lines under normoxia, but high levels can be induced by hypoxia or anoxia to generate a moderately alkaline intracellular pH (pHi) and an increasingly acidic extracellular pH (pHe) which are favorable for tumor cell growth and invasiveness [17]. Genetic depletion of CAIX in some carcinoma cells and human cancer xenografts reduces or attenuates primary tumor growth and inhibits metastasis formation [18, 19]. Unlike HIF-1, CAIX is a transmembrane protein, allowing efficient access by targeting and therapeutic agents. Therefore, CAIX constitutes a promising diagnostic biomarker of hypoxic tumor regions and an enticing target for augmenting response to the standard radiotherapy and chemotherapy. Correlation of its overexpression with poor response to classical chemotherapy and radiation makes CAIX an attractive molecular target for anticancer drug development [13]. CAIX-specific therapeutic modalities, monoclonal antibodies (mAb) and small molecule inhibitors, are being exploited to directly target the catalytic activity of CAIX for enhanced treatment efficacy [20].

Convenient properties of anisotropic GNRs have created strong interest in the development of antibody-targeted nanorods for a number of biomedical applications, including photothermal treatment combined with antibody therapy. By conjugating GNRs with anti-CAIX mAb, we expect to be able to ferry more GNRs to the most desirable location within tumors - that is, hypoxic areas that are more resistant to standard therapies. Focal targeting of these areas can address a source of treatment resistance more effectively than passive targeting of the entire tumor. In this study, we successfully fabricated a stable GNR/anti-CAIX nanoconstruct and tested its binding affinity and specificity to CAIX-positive cancer cells. We used photothermal ablation to document therapeutic efficacy *in vitro* and *in vivo*, demonstrating that this GNR/anti-CAIX nanoconstruct, combined with NIR laser offers a viable new photothermal intervention to combat tumor hypoxia.

## RESULTS

### Characterization of GNRs and GNR conjugates

**Figure 1:**
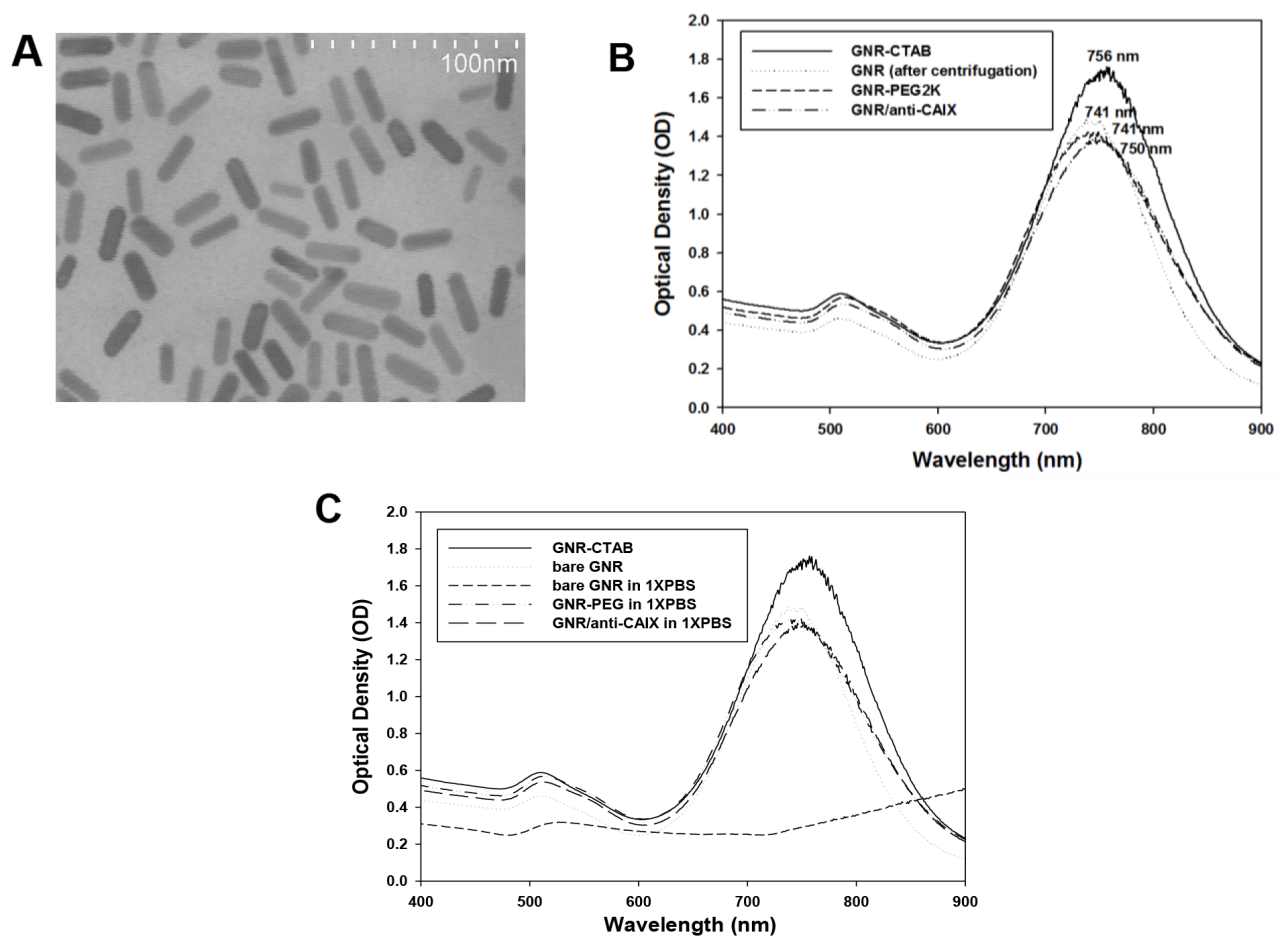
Characterization of GNR **(A)** TEM image of GNR. **(B)** UV-Vis spectra of GNR at each step of conjugation. **(C)** UV-Vis spectra of different GNRs after overnight incubation in 1x PBS. Bare GNR crashed while GNR-PEG and GNA/anti-CAIX were stable.

TEM revealed rod-like GNRs with lengths of ∼30 nm and diameters of ∼10 nm (Figure 1A). Zetasizer measurements confirmed the length of the GNRs at ∼30 nm and their zeta potential of 60 mV (with cetyl trimethylammonium bromide, CTAB coating). The UV-Vis spectra of GNR-CTAB exhibited two prominent surface plasmon resonance peaks, one in the visible range at ∼510 nm and another more prominent peak at ∼760 nm (Figure 1B). A bifunctional polyethylene glycol (PEG), ortho-pyridyldisulfide-polyethylene glycol 2000-N-hydroxysuccinimide ester (OPSS-PEG2K-SVA), was used to conjugate GNRs to anti-CAIX [1, 12, 21]. Compared with the GNR-CTAB and bare GNR (after centrifugation), shifts and changes of the NIR absorption, hydrodynamic diameter, and zeta potential of conjugated GNRs confirmed the success of each surface modification step (Table 1). A 15 nm blue shift of the longitudinal peak without peak broadening was noted with GNR-PEG compared to GNR-CTAB. Conjugation of anti-CAIX mAb results in a further 9 nm red shift of the longitudinal peak compared to the GNR-PEG.

**Table 1:** Size and zeta potential of GNRs, PEG-coated, and antibody conjugated GNRs

Surface ligands	Hydrodynamic diameter (nm)	Zeta potential (mV)
CTAB	32.7 ± 2.5	68.1 ± 9.1
After centrifuge	24.7 ± 0.3	55.5 ± 3.4
PEG-2K-SH protecting	32.0 ± 2.1	23.1 ± 3.1
OPSS-PEG-anti-CA IX	34.9 ± 1.2	9.2 ± 1.3
PEG-5K-SH final protecting	47.6 ± 1.6	4.1 ± 0.6

The observed spectral shifts can be attributed to changes in the refractive index of the nanorods’ surrounding environment [22]. The short-term stabilities of GNR, GNR-PEG and GNR/anti-CAIX were tested in 1X PBS buffer overnight, with optical spectra obtained afterward. As Figure 1C shows, the “as-made” GNR-CTAB and bare GNR had peak absorption at 755 nm and 745 nm, respectively. Overnight incubation in 1X PBS led to bare GNR forming aggregates and losing their peak at 745 nm, whereas GNR-PEG and GNR/anti-CAIX remained stable with preservation of their absorption peaks near 750 nm. These results suggested that the conjugated GNRs will be stable when exposed to blood, which had similar ionic strength as 1X PBS, and are therefore suitable for preclinical studies on laboratory animals.

### Quantification of antibodies per nanorod

The binding affinity of the GNR/anti-CAIX to CAIX protein and the numbers of antibodies per GNR particle were evaluated by using sandwich enzyme-linked immunosorbent assay (ELISA) (Supplementary Figure 1). Both the anti-CAIX mAb and the GNR/anti-CAIX conjugate showed increased binding to protein CAIX with increasing concentration, while little binding was found between GNR-PEG and the protein. The calibration linear curve for absorbance vs. anti-CAIX concentration showed an R^2^ of 0.997, and the curve of absorbance vs. GNR/anti-CAIX concentration was also linear with an R^2^ of 0.999. Based on these curves, a linear relationship was assessed for GNR/anti-CAIX OD vs. anti-CAIX mAb concentration (Supplementary Figure 2), and from the resulting equation we calculated that the number of anti-CAIX per GNR to be 5.0 ± 0.4, a value similar to previous findings [23]. In a preclinical study the therapeutic efficacy of an anti-CAIX mAb conjugated drug with an average drug-to-antibody ratio of n=∼4 showed a positive correlation with tumor CAIX expression level determined by immunohistochemistry and ELISA in tumor models [24].

### High specific cellular uptake of GNR/anti-CAIX in cells

**Figure 2:**
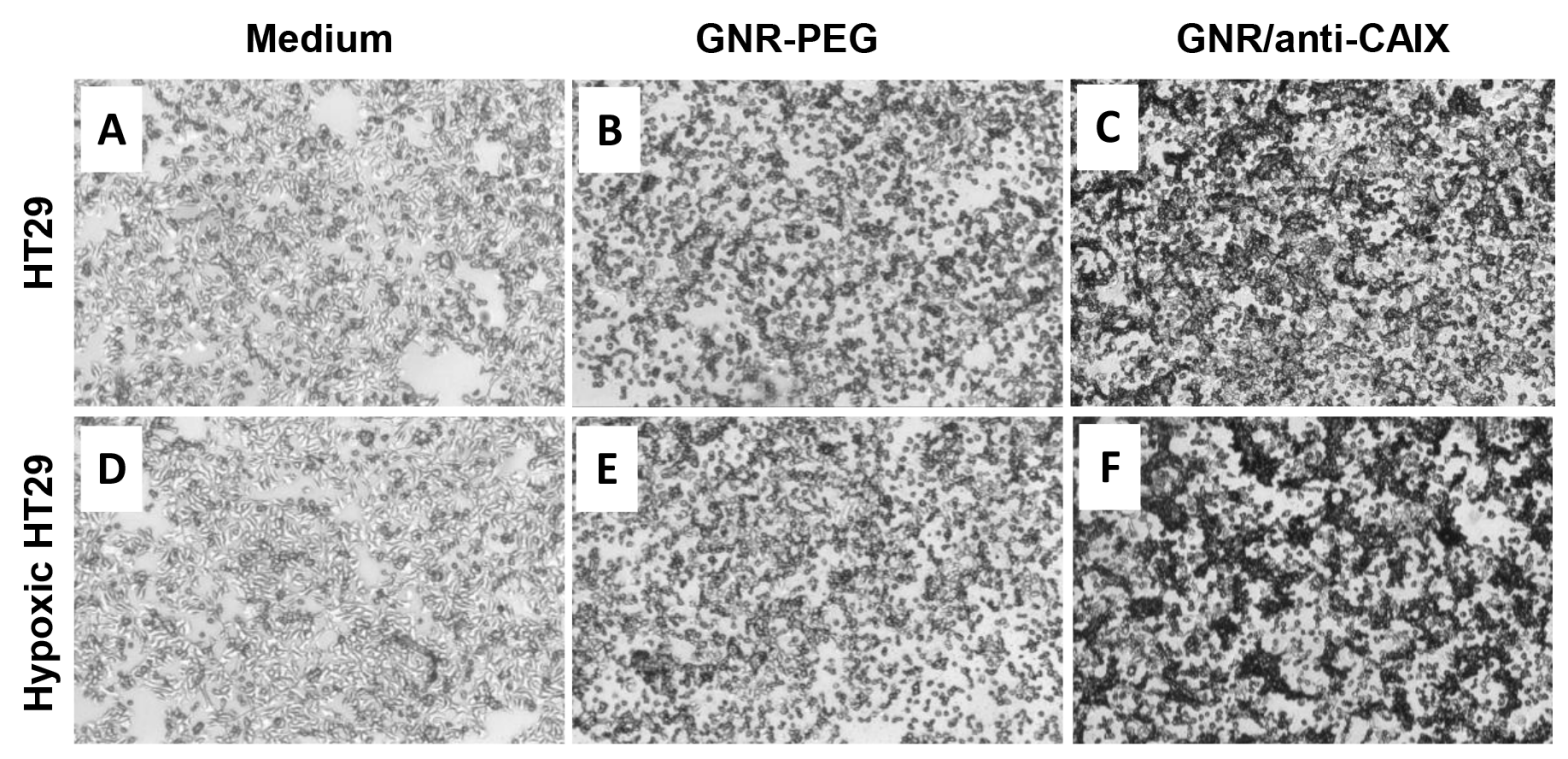
Silver staining of fixed HT-29 cells Incubated with medium only **(A** and **D)**, GNR-PEG (0.5 OD at 760 nm) in medium **(B** and **E)**, and GNR/anti-CAIX (0.5 OD at 760 nm) in medium **(C** and **F)** for 2 h. A, B and C were normal HT-29 cells; D, E and F were HT-29 cells in the CoCl_2_ induced hypoxic status.

Correlation of the uptake efficacy of the GNR/anti-CAIX with expression level of CAIX protein was assessed using both normoxic and CoCl_2_-induced hypoxia-mimetic conditions in HT29 cells. Co^2+^ replaces iron in the O_2-_sensing heme proteins, locking them into the “deoxy” conformation to induce expression of hypoxia-sensitive genes, in a manner similar to that observed with hypoxia [25]. Maximum uptake via receptor-mediated endocytosis of similarly sized PEGylated gold nanoparticles coated with ligands were reported at 2 h along with a fixed fraction of nanoparticles exocytosed [26, 27], therefore this time point was used for our cellular uptake studies. Silver enhancement was utilized to probe GNRs in cells by depositing metallic silver on the surface of GNRs until they were visible on bright field microscopy as black spots. The protein expression of CAIX of HT29 cells was upregulated significantly in response to CoCl_2_ treatment [28]. So as Figure 2 shows, a much higher uptake of gold was found in HT29 cells treated with GNR/anti-CAIX in contrast with GNR-PEG and medium control, indicating efficient targeting and successful conjugation. Also, hypoxic status did increase intracellular accumulation of GNRs treated with GNR/anti-CAIX at 2 h, which is in agreement with previous studies by showing a positive correlation between CAIX conjugates binding and expression of CAIX in human carcinoma cells [29, 30]. Hyperspectral dark field microscopy imaging also confirmed similar findings with the GNR/anti-CAIX group showing strong and specific binding to HT29 cells compared to the control group, while the GNR-PEG group only showing non-specific binding (Supplementary Figure 3). In addition, immunoconjugate specificity of GNR/anti-CAIX in cells has also been demonstrated by significant differences in binding capabilities between CAIX-positive HT29 cells and CAIX-negative NIH 3T3 cells, in which only HT29 cells labeled with GNR/anti-CAIX showed focal cellular black deposits, while none was observed in the NIH 3T3 cells labeled with GNR/anti-CAIX after 2 h (silver staining images as shown in Supplementary Figure 4).

### NIR photothermal treatment *in vitro*

**Figure 3:**
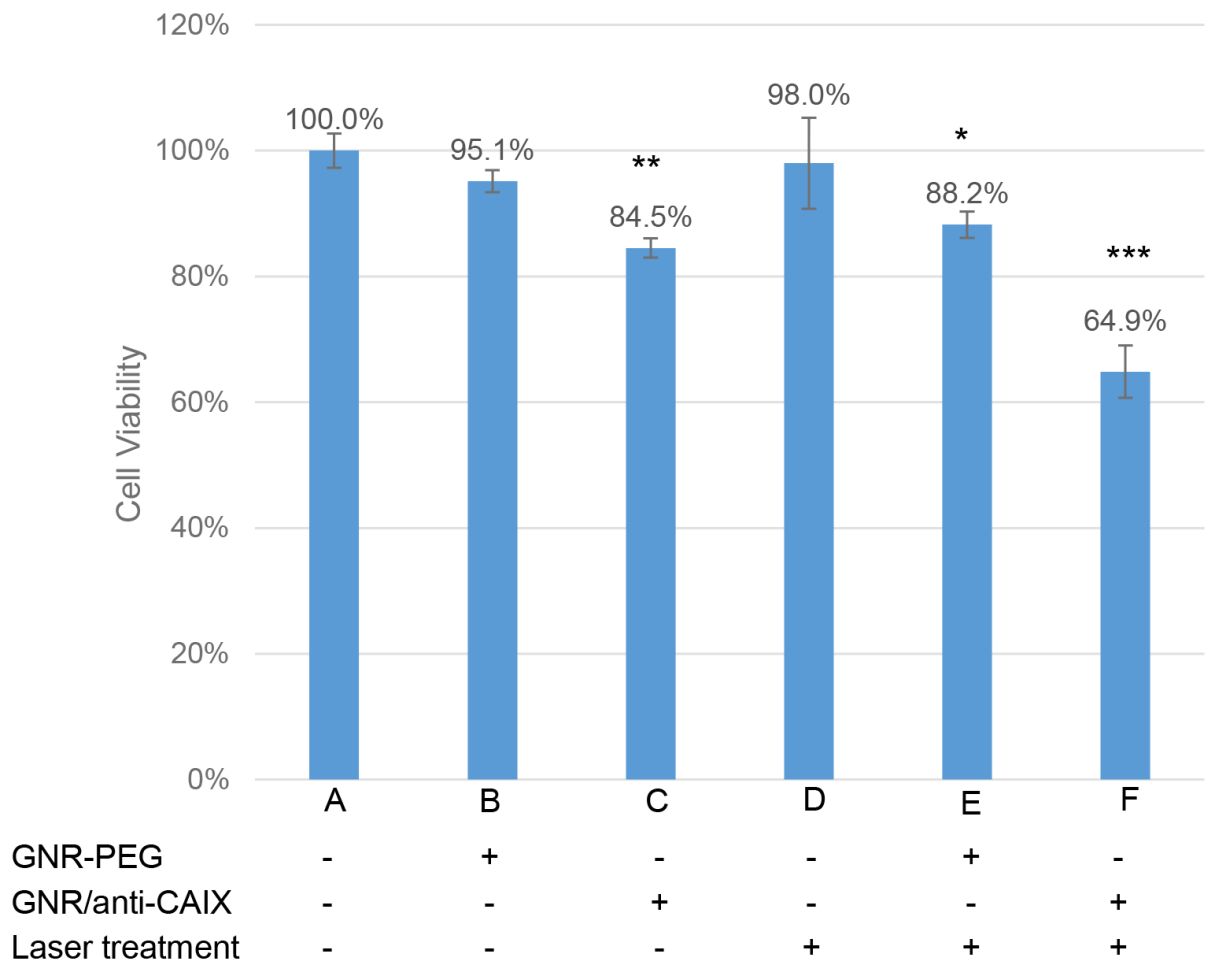
The survival percentage obtained for the HT29 cells after different treatment regimens No treatments but only medium (A) were applied as the control group which was used to evaluate other parameter on cell lethality (^*^p < 0.05, ^**^p < 0.01, ^***^p < 0.001). The data were expressed as mean ± SEM.

Having demonstrated hypoxia-specific accumulation of GNR/anti-CAIX in cells, we then studied if this could enhance photothermal effects in cells. As shown in Figure 3, HT29 cells incubated with GNR-PEG (0.5 OD at 760 nm) decreased little in viability as measured by MTT assay (B), while treatment with GNR/anti-CAIX (0.5 OD at 760 nm) alone increased cell death by 15.5% (P<0.01) compared to control cells (C). This antibody-dependent cell cytotoxicity (ADCC) may worked through directly targeting the catalytic domain of CAIX, disrupting the catalytic activity of the enzyme, including pH regulation and thus targeting its tumorigenic functions [31]. Epitopes of some anti-CAIX mAb fragments have showed a direct and prompt inhibition upon CAIX of human renal carcinoma cells in spheroid cultures [32]. Sole treatment of anti-CAIX mAbs in HT29 colorectal xenografts affectivity limited tumor growth after cell inoculation [33]. Moreover, the PG domain-specific G250 mAb and its fragments have been developed as CAIX-specific immunological tools for an adjuvant therapy against recurrence of renal cell carcinoma in patients [34]. The anti-CAIX mAb GT12 used in our study also binds to linear repetitive epitope in the PG region of native CAIX. Merely laser irradiation (12 W/cm^2^ for 2 mins) caused little cytotoxicity (Figure 3D). In contrast, laser irradiation in the presence of GNR/anti-CAIX conjugates significantly reduced the viability of HT29 cells to 64.9±9.3% (P<0.001) compared to GNR/anti-CAIX alone (P<0.01) and GNR-PEG with radiation treatment (P<0.05) (Figure 3F and 3E). Note that the laser spot is about 5 mm in diameter, which is about 60% of the growth surface in a well (96-well plate), therefore not all the cells in a well were directly treated with laser.

**Figure 4:**
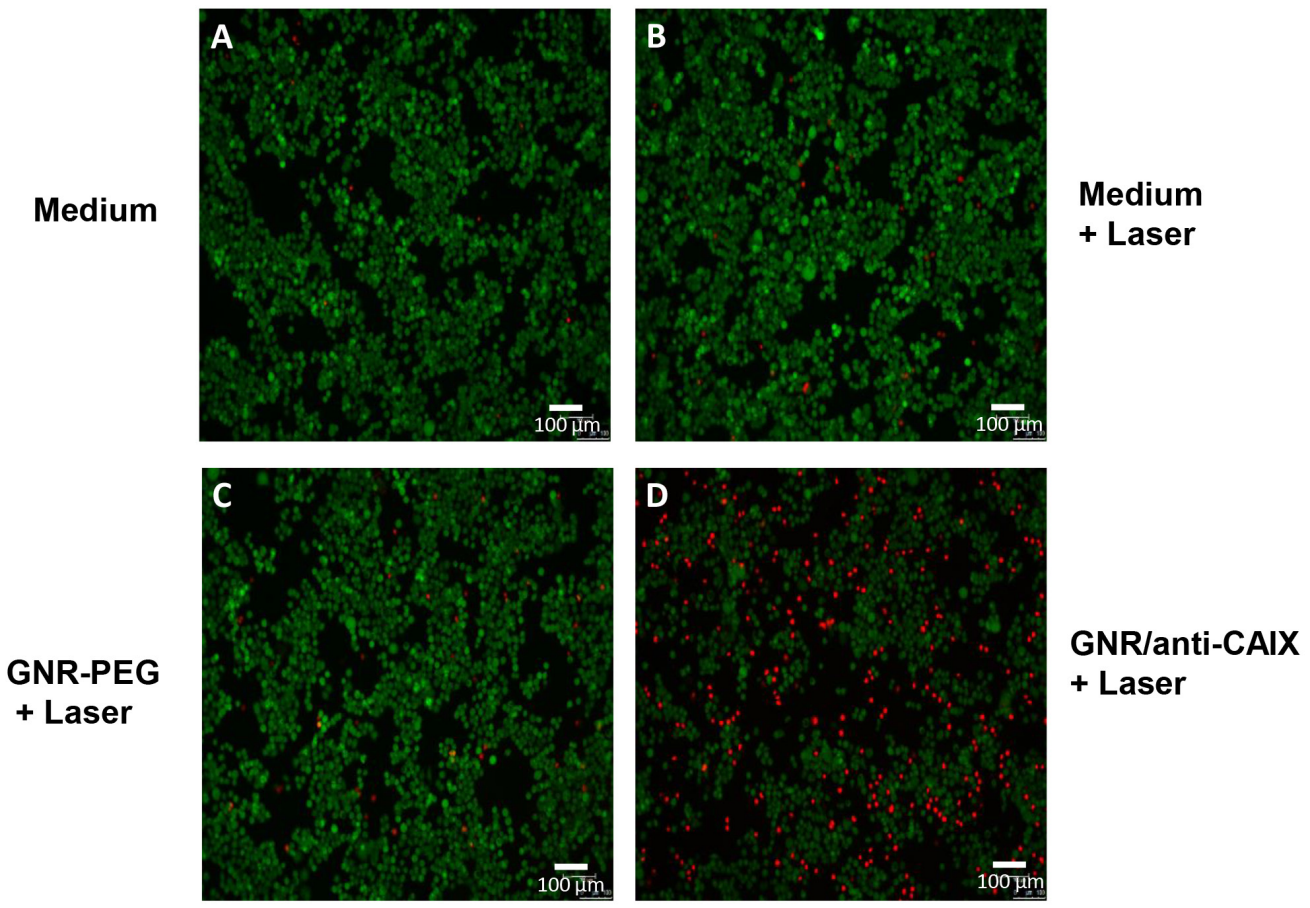
HT29 cell viabilities after photothermal treatment were imaged by fluorescence microscope under double staining by calcein AM and Propidium Iodide after an NIR laser irradiation (12 W/cm^2^) for 2 min Viable cells were in green and dead and dying cells were in red. **(A)** cells in growth medium, **(B)** cells treated with laser, **(C)** cells treated with GNR-PEG and laser, and **(D)** cells treated with GNR/anti-CAIX and laser.

Visual analysis of the cell viability was performed by double staining HT29 cells with calcein AM (485 nm excitation, 535 nm emission) and propidium iodide (PI, 530 nm excitation, 620 nm emission). The assay is based on the conversion of the cell permeant non-fluorescent calcein AM dye to the fluorescent calcein dye by intracellular esterase activity in live cells (green), while PI is membrane-impermeant and only intercalates its fluorescence with nucleic acids of dead cells (red). As Figure 4 shows, irradiating cells with up to 12 W/cm^2^ for 2 min caused significant cell death upon treatment with GNR/anti-CAIX, while the magnitude of cell death was similar in the GNR-PEG treated group and the control (medium treated) group, which suggested that retention of GNRs inside the cells via anti-CAIX mAb conjugation mediates efficacy of photothermal ablation. Note that we used a low laser power (temperature increase was low) which was further dissipated by the presence of media. Consequently, complete destruction of cells within the laser field was not expected and was not observed.

### Tumor and organ uptake

**Figure 5:**
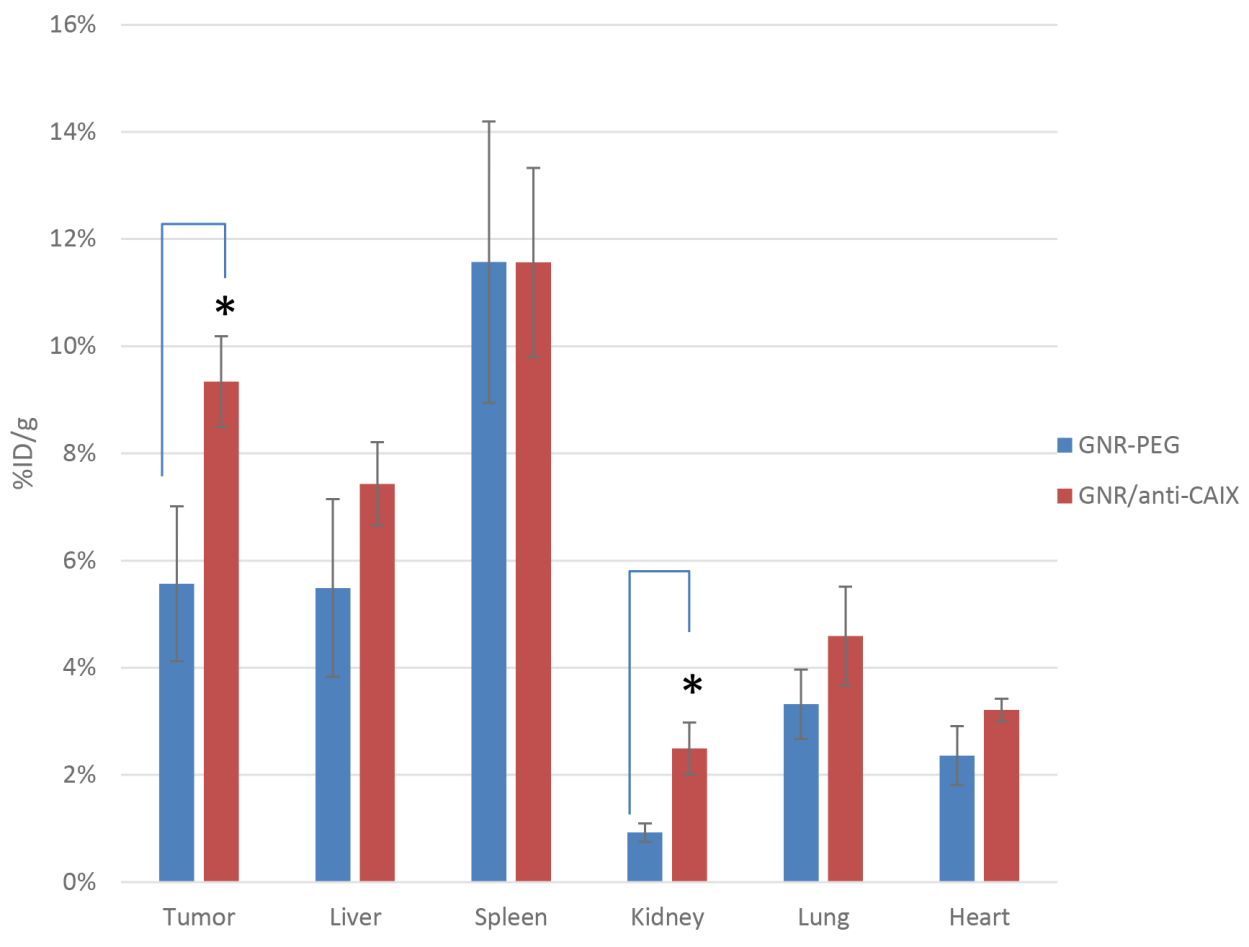
Comparison of gold content in tissues by ICP-MS 24 h after intravenous administration of targeted (GNR/anti-CAIX) and untargeted (GNR-PEG) GNRs in HT29-tumor-bearing mice (n=4 for both groups) The uptake of GNR/anti-CAIX was significantly higher than GNR-PEG in xenograft tumor (^*^p<0.05). The biodistribution in other organs was similar for both groups except higher uptake in the kidney for GNR/anti-CAIX.

As a prelude to investigating the *in vivo* photothermal ablation efficacy of the constructs, we first performed a detailed biodistribution analysis of not only individual organ and tumor accumulation of gold but also the geographical distribution of GNRs in tumor hypoxia. The optimal intratumoral accumulation of similarly sized PEGylated GNRs was observed at 24 h post injection (p.i.) in several previous studies in mouse xenograft models [35–37]. This time point was used for subsequent NIR photothermal treatments *in vivo*. Swiss Nu/Nu mice harboring subcutaneous HT29 xenografts were administered 100 μL of GNR-PEG or GNR/anti-CAIX intravenously and the tumors and normal organs were harvested 24 h later. As shown in Figure 5, the largest fractions of gold accumulation determined by ICP-MS were observed in the liver and spleen, consistent with the foreign body phagocytic activity of resident macrophages in these organs, the Kupffer cells of the reticuloendothelial system in the liver and the macrophages and B cells of the mononuclear phagocytic system in the spleen [38]. The relatively high uptake of GNRs in the tumor at 24 h p.i. is mainly due to the long circulation time of PEGylated GNRs and the enhanced permeability and retention (EPR) effect. Notably, the gold accumulation was higher in the GNR/anti-CAIX group (9.3 ± 0.8% ID/g), than the GNR-PEG group (5.6 ± 1.4% ID/g), indicating efficient targeting as seen with preferential accumulation of radiolabeled anti-CAIX mAb in xenografted HT29 tumor at 24 h [39–41]. Surprisingly, there was greater accumulation of gold in the kidney in the GNR/anti-CAIX group (2.5 ± 0.5% ID/g) compared to the GNR-PEG group (0.9 ± 0.2% ID/g). This may be due to size-dependent entrapment of in the corpuscles of the kidney mesangium [42] and/or preferential uptake of anti-CAIX mAb by the kidney at 24 h [41, 43]. No difference in gold accumulation in the lung and heart was noted between GNR/anti-CAIX and GNR-PEG.

### Location of GNRs in areas of tumor hypoxia

**Figure 6:**
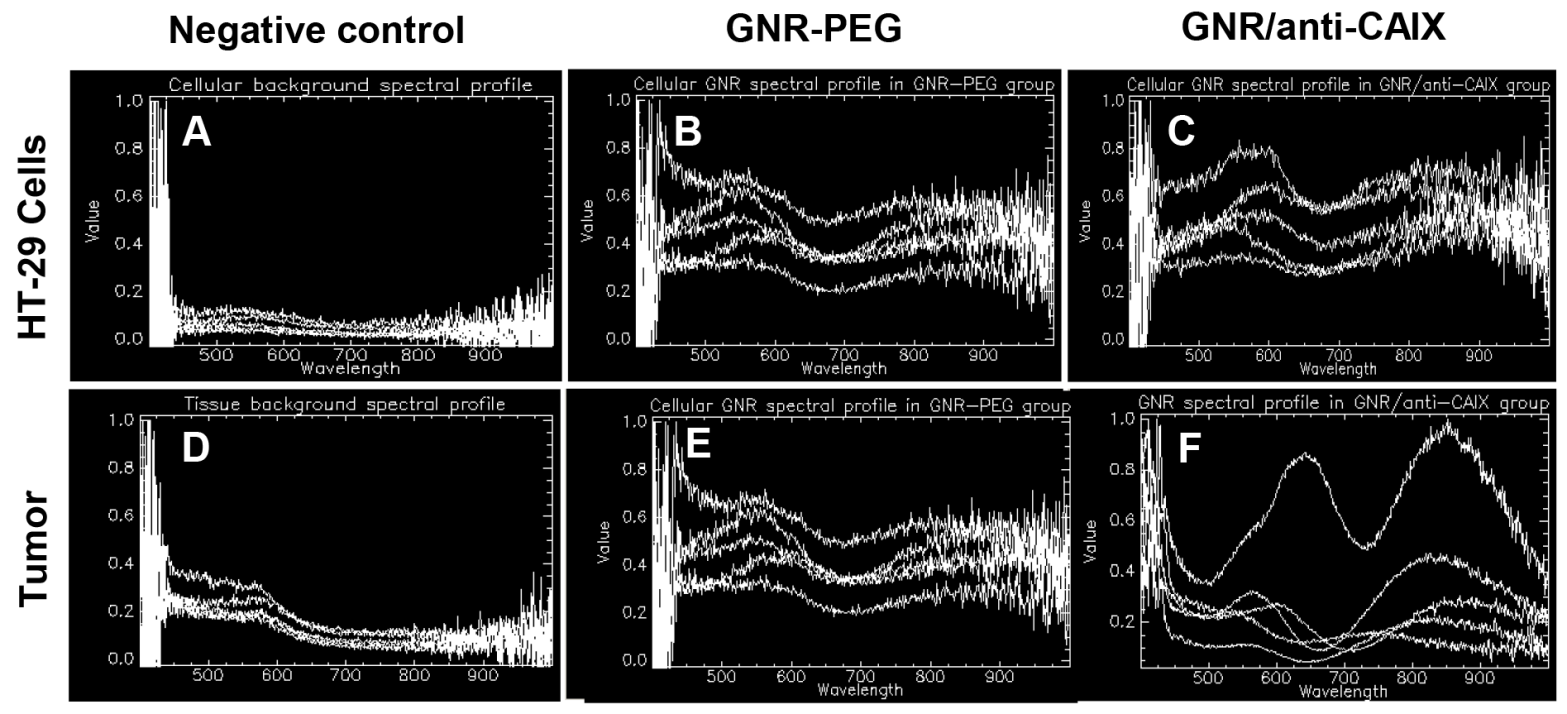
Hyperspectral imaging of GNRs exhibited unique spectral profiles in HT-29 cells and tumor **(A, D)** Background spectral profiles of control cells and control tumor without GNRs; **(B, E)** GNR-PEG in cells and tumor, respectively; **(C, F)** GNR/anti-CAIX in cells and tumor, respectively. Spectral shifts of GNR/anti-CAIX suggesting more complex interactions between cells and tumor tissues and GNR/anti-CAIX compared to GNR/PEG.

**Figure 7:**
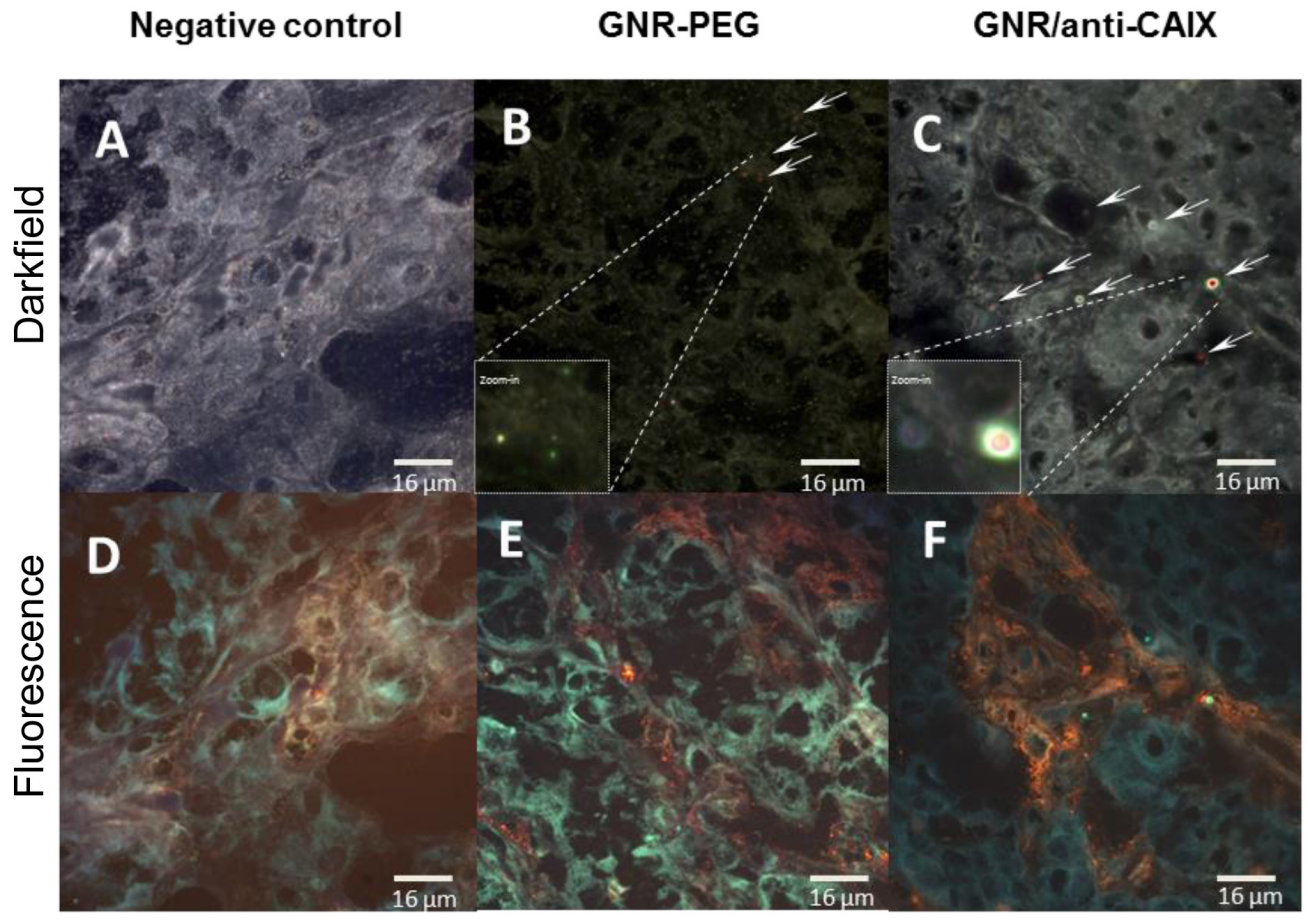
Representative 60x hyperspectral dark field images (A-C) and corresponding immunofluorescence (orange) images of tumor hypoxia characterized by pimonidazole uptake (D-F) **(A, D)**: negative control tissue; **(B, E)**: GNR-PEG; **(C, F)**: GNR/anti-CAIX. GNRs identified by hyperspectral analysis were mapped and marked with arrows and red color in darkfield images. Abundant and aggregated red pixels were found within hypoxic areas of GNR/anti-CAIX treated tumors compared to GNR-PEG treated tumors.

Although the biodistribution study confirmed greater tumor accumulation of targeted GNRs than nontargeted GNRs, this provides neither spatial information on the location of targeted GNRs relative to areas of hypoxia nor the degree of interaction of GNRs within tissue. Therefore, we analyzed tumor tissue sections by overlaying immunofluorescence images of hypoxic areas identified by pimonidazole accumulation and GNR location by hyperspectral imaging. The characteristic spectral profiles of the tumor matrix itself were first identified by enhanced dark field microscopy with hyperspectral imaging of tumors from animals treated without GNRs (Figure 6D, 7A, and 7D). After subtracting this background from tumors from animals treated with GNRs, the distinct dual absorbance peak spectra of GNRs (Figure 6E for GNR-PEG and Figure 6F for GNR/anti-CAIX that was spectrally shifted) was used to map their locations with tumors. In parallel, *in vitro* spectral profiles were also obtained of HT29 cells alone and in the presence of GNR-PEG and GNR/anti-CAIX (Figure 6A, 6B and 6C). Furthermore, tandem fluorescence imaging of fluorescently-labeled pimonidazole staining to identify areas of physical hypoxia and overlay of the hyperspectral image with the fluorescence image facilitated semi-quantitative comparison of the relative amounts of GNRs in hypoxic areas of histological sections. First, we observed a spectral shift toward higher wavelengths in the GNR/anti-CAIX group suggesting agglomeration of GNRs [44, 45], possibly due to endocytosis and aggregation in endosomes and lysosomes [46, 47]. Aggregation can be induced by coated anti-CAIX mAb proteolysis and the low pH environment of the endosome (pH 5.5) and lysosome (pH 4–5) [45]. Next, we observed more right-shifted and aggregated GNRs on the dark-field image (Figure 7C) of GNR/anti-CAIX treated tumors than GNR-PEG treated tumors (Figure 7B) confirming greater accumulation and internalization (with consequent aggregation) of GNRs in this group. Consistent with the notion that CAIX expression is more prominent in hypoxic areas [41] and correlates well with pimonidazole uptake [48], pimonidazole-positive areas of tumors harbored more GNRs in the targeted GNR/anti-CAIX group (Figure 7F) than in the untargeted GNR-PEG group (Figure 7E), where they were more randomly dispersed. No remarkable existence and well distribution of GNRs within hypoxia were observed in Figure 7C and 7F owing to ultrathin histological sections and aggregates of nanoparticles. Also, it is likely that the hyperspectral imaging was more sensitive at detecting agglomerated particles than individual 30nm-long GNRs which may be below the resolution limit of the microscope, obscured by backscatter from other sources or attenuated by tissue densities. Additional tissue section images have been included as Supplementary Figure 5. A new GNR functionalization strategy may facilitate a better distribution of GNRs in tissue hypoxia by changing ligand exchange method [49]. Nonetheless, taken together with the greater quantities of gold in the GNR/anti-CAIX group than the GNR-PEG group (on ICP-MS analysis), the geographic distribution results of the hyperspectral imaging study confirm the preferential accumulation of targeted particles in hypoxic areas of tumors.

### NIR photothermal treatment in tumor-bearing mice

**Figure 8:**
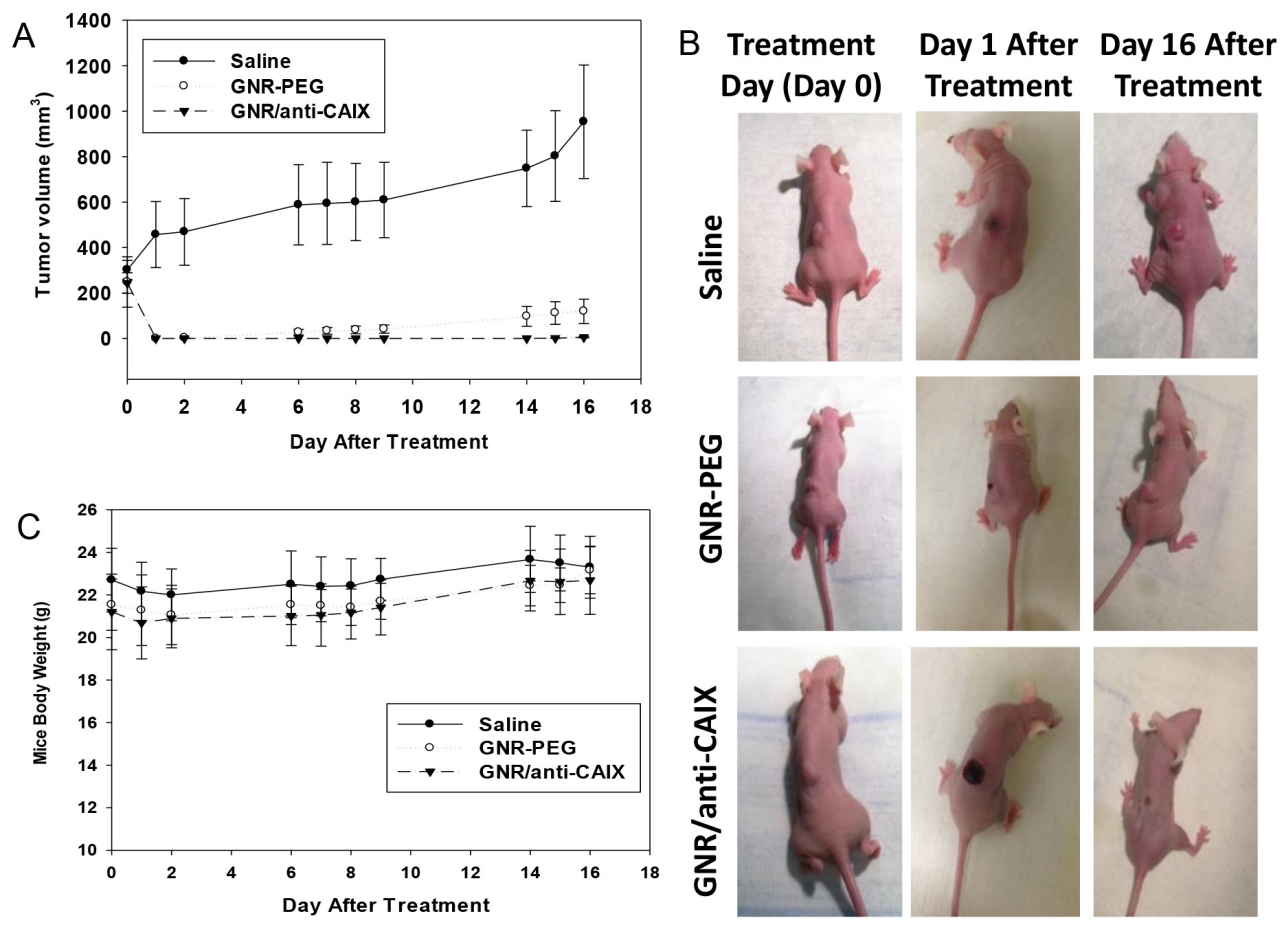
Photothermal ablation via near infrared irradiation of HT29 tumors 24-h after tail-vein injection of saline (n=3), GNR-PEG (n=5, OD=20) or GNR/anti-CAIX (n=7, OD=20) **(A)** Tumor volume plotted over time for all three groups. Data are presented as mean ± SEM. **(B)** Images of representative mice in each group prior to treatment, one day after treatment, and 16 days after treatment. No tumor regression in the saline-treated group; regression but recurrence of tumor in the GNR-PEG treated group; and complete tumor regression in the GNR/anti-CAIX treated group. **(C)** Body weight of mice in each group. No statistical difference between the three groups.

Having confirmed selective and preferential accumulation of GNR/anti-CAIX within areas of tumor hypoxia, we then evaluated the therapeutic effect of photothermal ablation of tumors laden with targeted and nontargeted GNRs *in vivo*. HT29-tumor-bearing nude mice injected intravenously with saline, GNR-PEG, or GNR/anti-CAIX were irradiated 24 h later with an NIR laser (760 nm, 12 W/cm^2^, 2-3 min). The temperature measured by the TRH Central probe within the tumor rapidly reached to ∼53°C within 2 to 3 min of NIR laser exposure on the skin surface above the tumor in GNR-PEG-injected mice and GNR/anti-CA IX-injected mice. As expected, the absolute temperature and rate of temperature increase were lower in the tumors of PBS (1X)-injected mice than that in the other two groups. Other photothermal studies using gold nanoparticles also suggested that temperature increases of 21.5°C upto 35°C achieved efficient tumor ablation without causing obvious toxicity [50–52]. Mice treated with saline showed uninhibited tumor growth after irradiation, with the mean tumor volume reaching 1000 mm^3^ 16 days later (Figure 8A). Tumors of mice injected with GNR-PEG or GNR/anti-CAIX, however, were completely ablated on the day of irradiation (day 1). Despite this complete ablation, tumors regrew slowly in the mice treated with GNR-PEG whereas they remained undetectable in the GNR/anti-CAIX group (Figure 8B). Notably, although the ablation scar was small in the saline-treated group, large in the GNR-PEG group and most prominent in the GNR/anti-CAIX group, body weights of mice in all groups remained largely unchanged indicating the lack of any overt acute toxicity (Figure 8C). Collectively, these findings demonstrate that GNR/anti-CAIX constructs can improve the antitumor effectiveness of NIR laser photothermal treatment while retaining a mild toxicity profile.

## DISCUSSION

In this study, we demonstrated that decoration of ∼30 × 10 nm GNRs with anti-CAIX antibodies permits preferential targeting of cells harboring cell-surface CAIX protein that then facilitates selective ablation of these cells upon NIR irradiation *in vitro*. When administered *in vivo*, these targeted constructs accumulate in greater quantities in tumors than untargeted GNRs and selectively sequester within hypoxic areas where their internalization in CAIX-overexpressing cells results in intracellular aggregation. Photothermal ablation of HT29 tumors in mice treated with GNR/anti-CAIX resulted in sustained and durable complete tumor regression whereas tumors treated with GNR-PEG showed an immediate regression but eventually regrowth, possibly due to the persistence of treatment-resistant hypoxic cells. A frequent source of puzzlement is the assumption that if even a small molecule like oxygen is unable to reach a hypoxic cell a larger nanoparticle would have no ability to reach this cell. However, hypoxia within a tumor is not strictly a function of inability of oxygen to diffuse far from the feeding vessel, but rather is a combination of diffusion-limited restriction of oxygen transport and consumption of oxygen by intervening cells between the vascular endothelium and deeper areas away from the vessel. Thus, nanoparticles are possible to penetrate to these depths from the tumor vasculature even if they are larger than oxygen molecules, as long as they are not consumed by intervening cells.

Treatment conditions in our study, including the threshold laser power density (12 W/cm^2^) and exposure time (2-3 min) at which GNR/anti-CAIX induced tumor regression were similar to some previous reports of *in vivo* NIR irradiation that used other NIR-absorbing gold nanostructures injected via tail veins, such as nanorods (2 W/cm^2^, 5 min) [2], nanoshells (4 W/cm^2^, 3 min) [1], and nanospheres (3 W/cm^2^, 5 min) [53]. The lowest NIR laser dose that produced tumor ablation with GNR that we could find was 0.9-1.1 W/cm^2^ with a 6-min-long irradiation [35]; however, the GNRs in that study were administered intratumorally. Notably, intravenous administration of 100 μL of 20 OD particles in our study compares favorably with 100 μL of 50-120 OD particles utilized in other studies [1, 2, 53]. Collectively, our findings, juxtaposed with previous studies, suggest that GNRs conjugated to anti-CAIX can be used as selective and efficient photothermal agents for tumor ablation using a low-energy, harmless NIR laser with brief exposure times that cause minimal damage to surrounding normal tissue. Our improved therapeutic efficacy could result from better targeting of GNR to tumor hypoxia via the guidance of anti-CAIX. Furthermore, eliminating the hypoxic fraction of tumor cells within tumors and forestalling recurrences via CAIX targeted nanoparticle approaches could be a potent adjunct to conventional therapies that often fail due to the recalcitrance of hypoxic cells to these therapies. Understandably, this approach to targeting tumors and overcoming treatment resistance can be applied broadly across all tumor types without the need for decorating GNRs with moieties homing onto tumor-specific epitopes that vary across tumor types. Therefore, this could be a turn-key class solution to the clinical challenge of hypoxic treatment resistance across multiple tumor types.

## MATERIALS AND METHODS

### Materials

Gold (III) chloride hydrate (HAuCl_4_), CTAB, sodium borohydride (NaBH_4_), L-ascorbic acid, silver nitrate (AgNO_3_), sodium sulfate (Na_2_S), and bovine serum albumin (BSA) were purchased from Sigma-Aldrich (St. Louis, MO, USA). CAIX recombinant protein and secondary goat anti-mouse IgG antibody (HRP), were purchased from Novus Biologicals (Litttleton, CO, USA). Mouse monoclonal CAIX antibody GT12 was purchased from GeneTex (Irvine, CA, USA). PEG 2000 thiol (PEG-2K-SH), PEG 5000 thiol (PEG-5K-SH) and bifunctional OPSS-PEG2K-SVA were purchased from Laysan Bio, Inc (Arab, AL, USA). Human colon adenocarcinoma cell line HT29 was purchased from the American Type Culture Collection (Manassas, VA). DMEM medium with 4.5 g/L glucose, L-glutamine & sodium pyruvate and Dulbecco’s phosphate-buffered saline 1X without calcium and magnesium (1X DPBS) were purchased from Corning (New York, NY, USA). Phosphate-buffered saline 1X without calcium and magnesium (1X PBS) was purchased from Hyclone laboratories, Inc (Logan, Utah, USA).

### GNR synthesis

GNRs were synthesized via the seed-mediated growth method [54]. Briefly, 250 μL of 10 mM HAuCl_4_ was dissolved in 7.5 mL of 10 mM CTAB solution by stirring, and 600 μL of ice-cold NaBH_4_ (10 mM) was quickly added to the solution; after 2 h at 30°C, 2- to 5-nm gold nanoparticles were formed as a seed solution. A growth solution was prepared by mixing 40 mL of CTAB (10 mM), 1.7 mL of HAuCl_4_ (10 mM), 250 μL of AgNO_3_ (10 mM) and 270 μL of L-ascorbic acid (100 mM), after which 840 μL of seed solution was added into growth solution for 30 min to form GNRs until 100 μL of 10 mM Na_2_S was added to stop the reaction. The final GNR suspension was characterized by an ultraviolet/visible wavelength (UV-Vis) spectrophotometer (U-0080D, Hitachi, Schaumburg, IL, USA) and dynamic light scattering/zeta potential analyzer (Nano-ZS Zetasizer, Malvern, Westborough, MA, USA). The size and shape of GNRs were examined by transmission electron microscopy (TEM). Samples were prepared by placing one drop of the GNR suspension on a 200-mesh copper grid with lacey carbon (SPI Supplies, West Chester, PA), and drying in a vacuum oven overnight, followed by imaging with a Hitachi S-4800-II transmission electron microscope (Hitachi High Technologies America, Inc., Dallas, TX).

### Preparation of GNR conjugates

**Figure 9:**
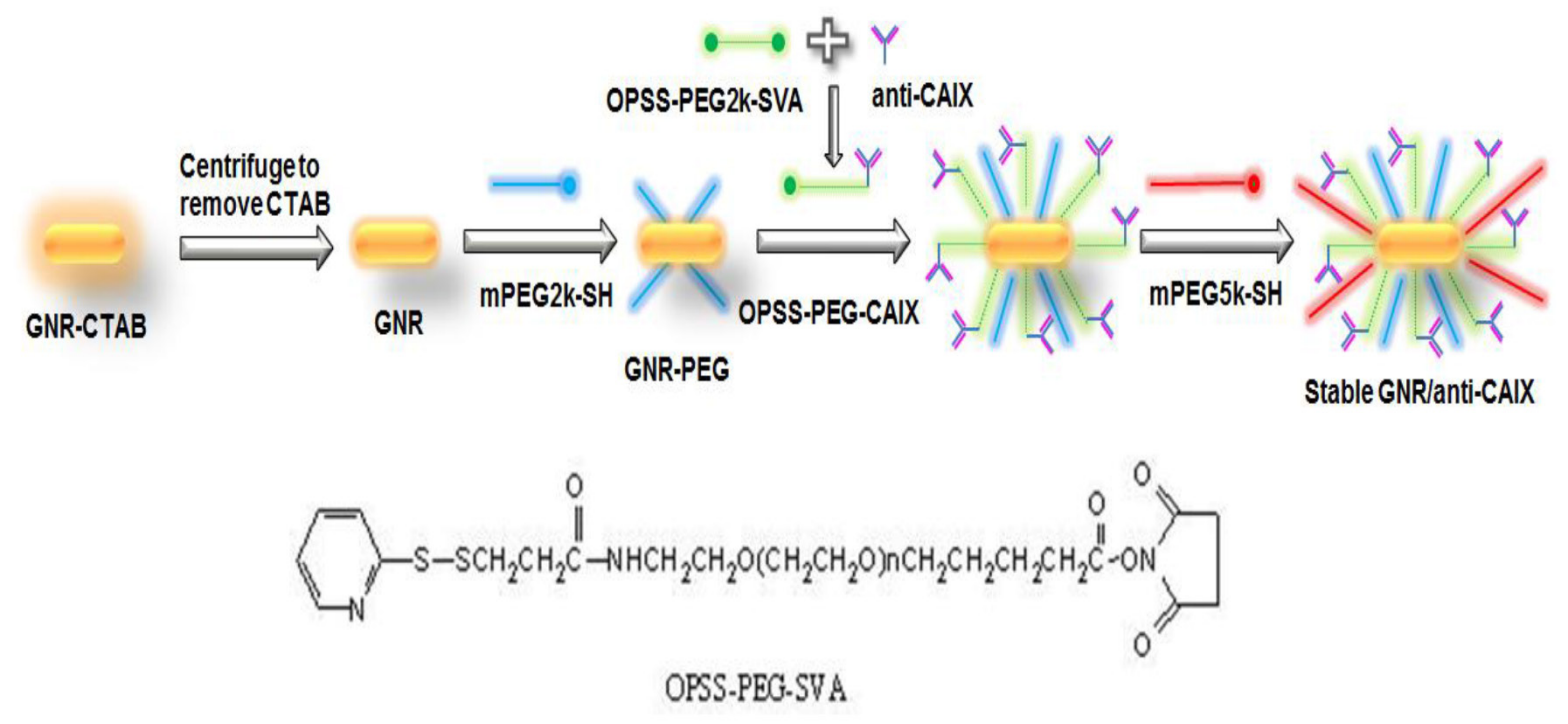
Preparation of hypoxia-targeted GNRs The conjugation of GNRs with anti-CAIX antibody via bi-functional crosslinker OPSS-PEG-SVA.

GNRs and anti-CAIX were conjugated in a five-step fashion as shown in Figure 9. First, anti-CAIX was conjugated to OPSS-PEG2k-SVA by mixing them at 1:1.5 molar ratios overnight at 4°C [12]. Second, the GNR suspension (5 mL, 0.5 OD at peak wavelength as made above) was obtained by centrifugation at 10,000 rpm for 30 min to remove the extra CTAB [55]. Third, the particles were semi-stabilized by adding a very small amount of PEG-2K-SH (1 μM). Fourth, 30 μL of the OPSS-PEG-CAIX was immediately added into the GNR suspension (50:1 molar ratio) and the mixture was placed on a shaker at room temperature for 1 h, allowing the OPSS group to conjugate to the gold surface of the particles. Finally, PEG-5K-SH was mixed with GNR/anti-CAIX at a molar ratio of 100,000:1 to backfill the surface of GNR and nearly neutralize the surface charge of GNR, thereby increasing their stability and circulatory half-life. The mixture was centrifuged at 10,000 rpm for 10 min to remove the free OPSS-PEG-CAIX and extra PEG-5K-SH, and the pellets were re-suspended in 1X PBS (pH 7.4). Changes in UV-Vis absorbance, size, and zeta potential were assessed at each step of the conjugation procedure with a UV-Vis spectrophotometer and Zetasizer as described in the preceding paragraph.

### Sandwich enzyme-linked immunosorbent assay (ELISA)

A sensitive sandwich ELISA was developed for determining the number of captured anti-CAIX per GNR (Supplementary Figure 1). CAIX protein was coated on 96-well plates at 0.1 μg/well. After incubation overnight at 4°C, the plates were washed with washing buffer (1X PBS with 0.05% Tween 20), and then blocked with blocking buffer (1X PBS with 2% BSA) at room temperature for 2 h. The blocking buffer was removed, and serial dilutions of anti-CAIX, GNR-PEG, or GNR/anti-CAIX (prepared with blocking buffer) were added in triplicate. The starting concentration of anti-CAIX was 0.11 μg/mL and GNR-PEG and GNR/anti-CAIX were of 5 OD at ∼760 nm. After 1 h of incubation at room temperature, the ELISA plates were washed five times with washing buffer. The binding affinity of anti-CAIX was detected by adding HRP, followed by 1 additional hour of incubation and five washes. At that time 100 μL of substrate solution was added to each well and the plates incubated for 10∼15 min in the dark. Finally, 100 μL of stop solution was added to each well and incubated for 15 min. The absorbance was then read at 450 nm with a DTX 880 multi-mode detector (Beckman Coulter, South Kraemer Boulevard Brea, CA). The resulting absorbance intensities were plotted against anti-CAIX concentrations to get a standard curve allowing calculation of the amount of anti-CAIX attached to GNR.

### Cellular uptake of coated GNRs

The treatment group of HT29 cells was cultured on Poly-D-lysine coated cover glass (NeuVitro) in wells under standard conditions and grown to ∼80% confluence, followed by treatment with cobalt (II) chloride (CoCl_2_) at a final concentration of 100 μM overnight to induce hypoxia [56], while the control group only exposed to growth medium throughout. Growth medium was removed and cells were rinsed three times with 1x DPBS (pH 7.2) before treatment with GNR-PEG (500 μL/well of 0.5 OD at 760 nm), GNR/anti-CAIX (500 μL/well of 0.5 OD at 760 nm) or media alone. After incubation for 2 h, the wells were rinsed three times with ice-cold 1x DPBS and chemically fixed with 2% glutaraldehyde in 1x DPBS at 4°C for overnight, then rinsed four times with deionized water. Both of silver staining and hyperspectral dark-field imaging were incorporated to measure cellular uptake of GNRs in HT29 cells. Silver enhancement of binding was performed with the Silver Enhancer kit (SE100, Sigma) according to the manufacturer’s protocol. After ∼5 min the development was stopped by washing with deionized water and 2.5% sodium thiosulfate solution. The intensity of the silver enhanced label was documented with a bright field microscope. The CytoViva Hyperspectral Imaging System (CytoViva Inc., Auburn, AL, USA) featuring enhanced dark field microscopy was used to locate GNRs in cell slides. Hyperspectral profiles were acquired using a Pixelfly camera and visualized using ENVI 4.8 software. To confirm the identity of GNRs, we used z-spectral profiles to create spectral libraries of each section containing GNRs and compared to that of the negative control. Then, spectra extracted from tissue slices that overlapped with the spectral profile of GNRs in solution with two absorption peaks at ∼510 nm and ∼ 760 nm (axial and longitudinal plasmon resonance peaks, respectively) were chosen as representative of GNRs in cells. The Spectral Angle Mapper Classification (SAM) procedure was used to mark pixels within images with characteristic GNR spectra.

### *In vitro* NIR photothermal laser treatment

The cellular response to treatment with GNR-PEG or GNR/anti-CAIX and laser irradiation was assess using the MTT and fluorescence-based cell viability assays. Briefly, 1×10^4^ HT29 cells in 100 μl of complete medium were seeded in a 96-well plate or 24-well plate and incubated overnight. Either GNR-PEG or GNR/anti-CAIX at a final concentration of 0.5 OD in culture treatment medium was added, with blank medium used as a control. After 2 h of incubation, cells were rinsed three times with DPBS to remove any free GNRs and replenished with fresh medium. An NIR laser, at a wavelength of 760 nm (to overlap the spectral absorption of the GNRs) with a spot-size of ∼5 mm in diameter and power density of 12 W/cm^2^ was used to irradiate each well for 2 min. 20 μL of MTT solution (5 mg/mL) was added to each well of 96-well plate and incubated for an additional 4 h, while 2.5 μM Propidium Iodide (PI, Life Technologies) and 3 μM calcein AM (Cell Biolabs) were added to 24-well plates to evaluate viability by fluorescence microscopy (DMI600 B; Leica). At the end of the incubation time, formazan crystals were dissolved by 200 μL dimethyl sulfoxide (DMSO) and the plate was placed on the orbital shaker for 10 min. Finally, the plates were read at 590 nm with a 630 nm reference using a DTX 880 multi-mode detector. The viability rate (%) of cells in different groups was calculated by the following formula: viability rate = (average absorbance of treated group/average absorbance of the control group) ×100%.

### Biodistribution study

Eight (8) six-week-old male Swiss Nu/Nu mice purchased from the in-house MD Anderson colony were inoculated by subcutaneous injection of ∼2 × 10^6^ HT29 cells in the right thigh, according to a protocol approved by the IACUC at MD Anderson Cancer Center. Mice were injected with either 100 μL of GNR-PEG (n=4,20 OD) or 100 μL of GNR/anti-CAIX (n=4,20 OD) once the mean tumor size reached a diameter of ∼7mm. The animals were euthanized at 24 h p.i., and tumors and organs (liver, kidney, spleen, heart and lung) were collected for assessment. The tumors and organs were washed in iced 1x PBS buffer, frozen, and then lyophilized overnight. Each dried tissue sample was weighed, dissolved in 1 mL of aqua regia then diluted with 9 mL of 2% hydrogen chloride (HCl) for a total volume of 10 mL. Tissue debris was removed using 0.1 μm syringe-driven filters (Millex-VV from EMD Millipore). The gold content was analyzed by ICP-MS (Varian 810-MS, Agilent, Santa Clara, CA) at the Inductively Coupled Plasma Mass Spectrometry Analytical Lab of University of Houston. Results were expressed as a percentage of the injected dose per gram (%ID/g).

### Histological processing and imaging

As previously described, Swiss Nu/Nu mice were xenografted with HT29 tumors, following an animal protocol approved by the IACUC at MD Anderson Cancer Center. Three (3) mice each were intravenously injected with either 100 μL of GNR-PEG (20 OD) and 100 μL of GNR/anti-CAIX (20 OD) and one (1) mouse received saline alone when the mean tumor size reached ∼7 mm. At 24 h p.i. mice were then administered 2.5 mg pimonidazole (Hypoxyprobe, Burlington, MA, USA) intravenously and 1 h later administered 0.4 mg Hoechst 33342 (Sigma-Aldrich) intravenously, followed immediately by euthanasia. Tumors were excised and embedded in optimal cutting temperature compound for frozen section analysis. Four to five-micron sections were stained overnight (at 4°C) with anti-pimonidazole primary antibody (Hypoxyprobe, Burlington, MA, USA) diluted (1:50) in antibody diluent (Dakocytomation) followed by 1 h incubation (at 20°C) with Alexa Fluor 555 labeled anti-mouse secondary antibody (ThermoFisher, Waltham, MA, USA) using a 1: 100 dilution. GNRs in prepared tissue sections were identified by hyperspectral dark field imaging using the same parameter settings described in the cellular uptake study. The Alexa Fluor 555 fluorescence was captured by a Dage camera coupled with dual mode fluorescence module with appropriate excitation/emission filters.

### *In vivo* NIR photothermal laser treatment

Fifteen (15) 8-week-old female CR ATH HO nude mice (Charles River) with HT29 tumors engraftment, following an animal protocol approved by IACUC at Texas Southern University, were randomly assigned to one of three treatment groups when the mean tumor size reached ∼7 mm. Mice in all three groups were anesthetized and administered via tail vein with 100 μL of saline (n=3 mice), 100 μL of GNR-PEG (n=5,20 OD) or 100 μL of GNR/anti-CAIX (n=7,20 OD). GNRs were allowed 24 h after injection to accumulate in tumors, and then all tumors were irradiated with an NIR laser (Coherent, Santa Clara, CA, USA) through the skin surface, covering the tumors with power density of 12 W/cm^2^ and spot diameter of 10 mm for 2-3 min. Interstitial temperature in the HT29 tumors was measured with a TRH Central probe (Omega, Stamford, CN, USA). Laser exposure was immediately stopped if the temperature reached 60°C. Tumor sizes and body weights of the mice were measured and recorded for up to 16 days after laser treatment. Dimensions of the resulting tumors were measured with digital calipers, and tumor volumes were calculated as volume = length × width^2^ /2.

### Statistical analysis

At least three replicates for each experimental or control were performed per assay. All quantitative data were expressed as mean ± standard error of the mean (SEM). Statistical comparisons between groups were performed by student’s *t* test or one-way analysis of variance (ANOVA), as applicable, and two-sided p values of 0.05 or less were considered statistically significant.

## SUPPLEMENTARY MATERIALS FIGURES



## Abbreviations

ADCC: antibody-dependent cell cytotoxicity
BSA: bovin serum albumin
CAIX: carbonic anhydrase IX
CTAB: cetyl trimethylammonium bromide
ELISA: sandwich enzyme-linked immunosorbent assay
EPR: enhanced permeability and retention
GNRs: gold nanorods
HIF-1: hypoxia inducible factor 1
ICP-MS: inductively coupled plasma mass spectrometry
mAb: monoclonal antibodies
NIR: near-infrared
PBS: phosphate-buffered saline
PEG: polyethylene glycol
OPSS-PEG2K-SVA: ortho-pyridyldisulfide-polyethylene glycol 2000-N-hydroxysuccinimide ester pHe, extracellular pH
pHi: intracellular pH
p.i.: post-injection
PI: propidium iodide
TEM: transmission electron microscopy
UV-Vis: ultraviolet/visible wavelength
