# Supplementary material for: Hypoxia-targeted gold nanorods for cancer photothermal therapy
Source: Oncotarget. 2018 May 29;9(41):26556–71. doi: 10.18632/oncotarget.25492 (PMC5995181; doi:10.18632/oncotarget.25492)
Supplement: Supplementary file 1 [file oncotarget-09-26556-s001.pdf]

Hypoxia-targeted gold nanorods for cancer photothermal therapy

SUPPLEMENTARY MATERIALS

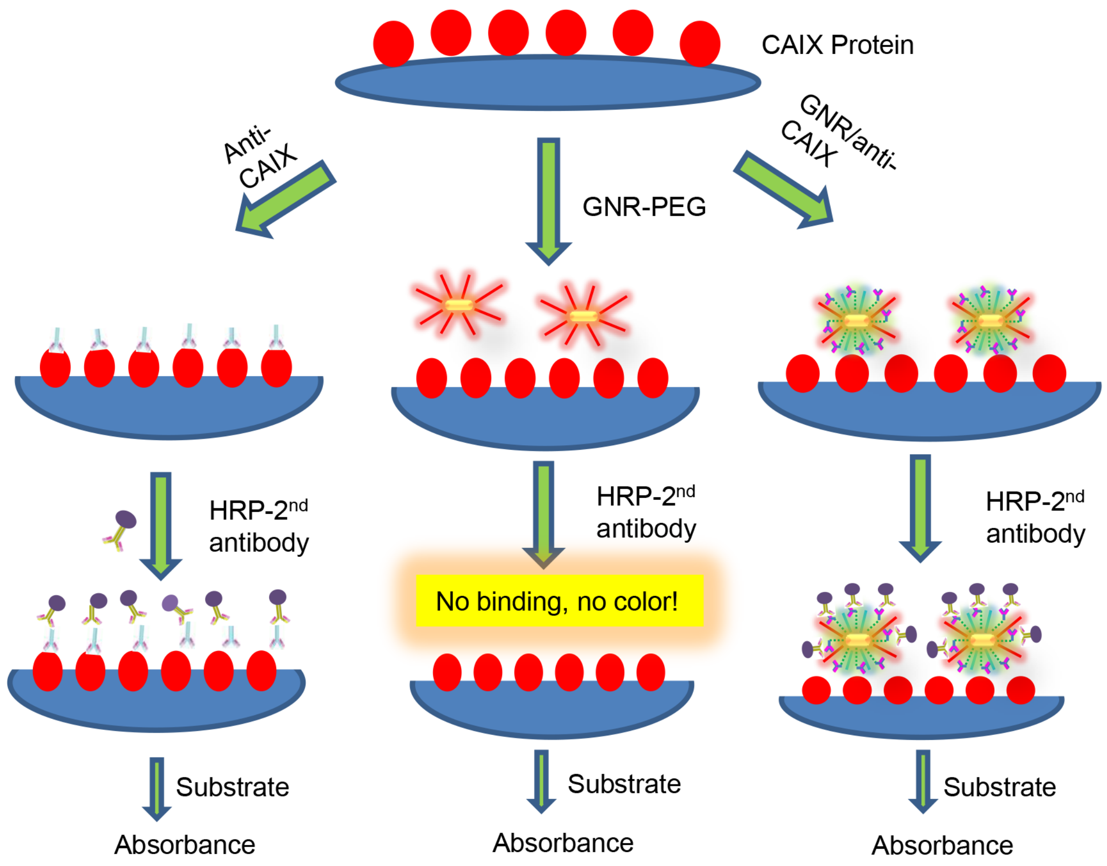

Supplementary Figure 1: Schematic representation of sandwich ELISA. The absorbance of HRP-2nd antibody is proportional to the amount of anti-CAIX, thereby allowing quantification of the number of anti-CAIX per GNR. GNR-PEG that lacks anti-CAIX does not bind the CAIX and therefore has no absorbance.

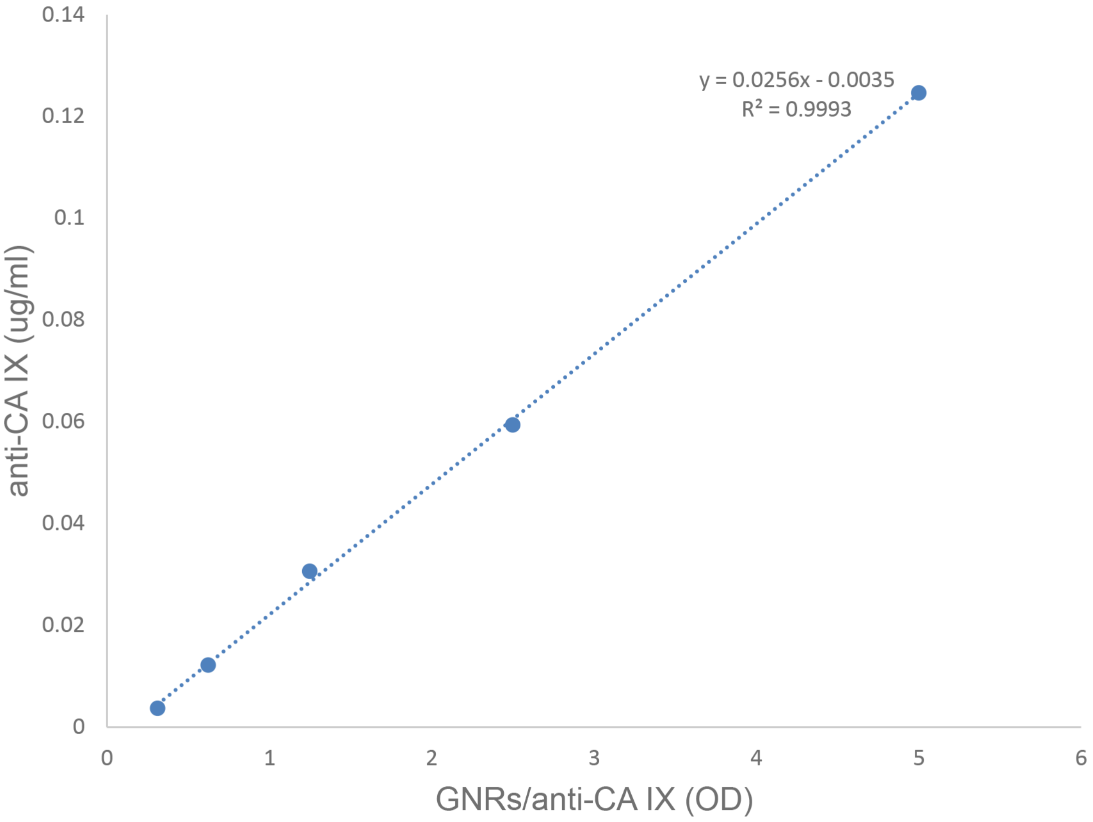

Supplementary Figure 2: Calibration curve of concentration of anti-CAIX vs. GNR/anti-CAIX OD.

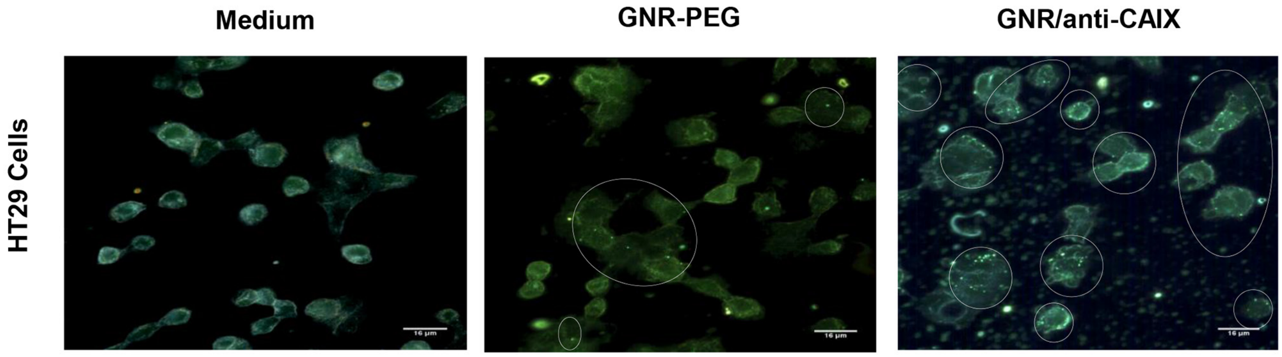

Supplementary Figure 3: Hyperspectral dark field microscopy images of fixed HT-29 cells incubated with medium only, GNR-PEG (0.5 OD at 760 nm) in medium, and GNR/anti-CAIX (0.5 OD at 760 nm) in medium for 2 h. Accumulation of GNRs with distinct spectral characteristics in cells is depicted by circles within the images.

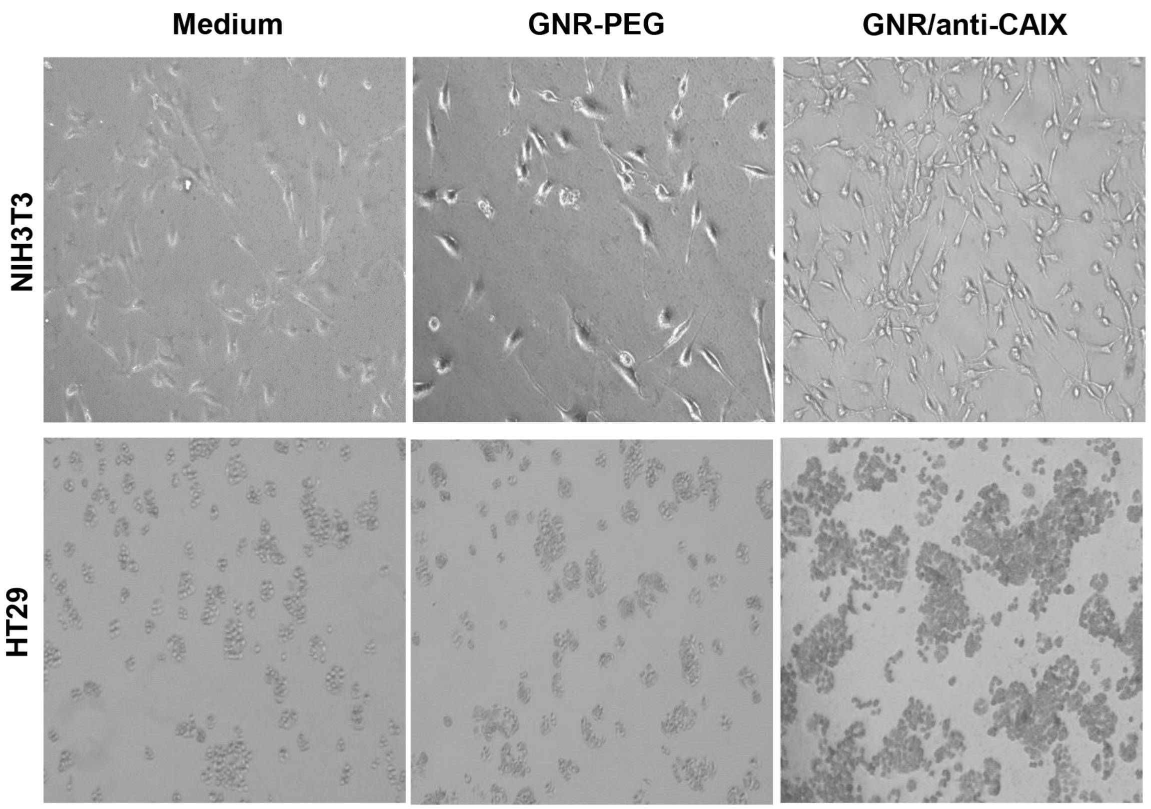

Supplementary Figure 4: Selective binding of GNR/anti-CAIX to HT29 cells. Silver staining of CAIX non-expressing NIH 3T3 cells (upper row) and CAIX overexpressing HT29 cells (lower row) after incubation with medium, GNR-PEG and GNR/anti-CAIX.

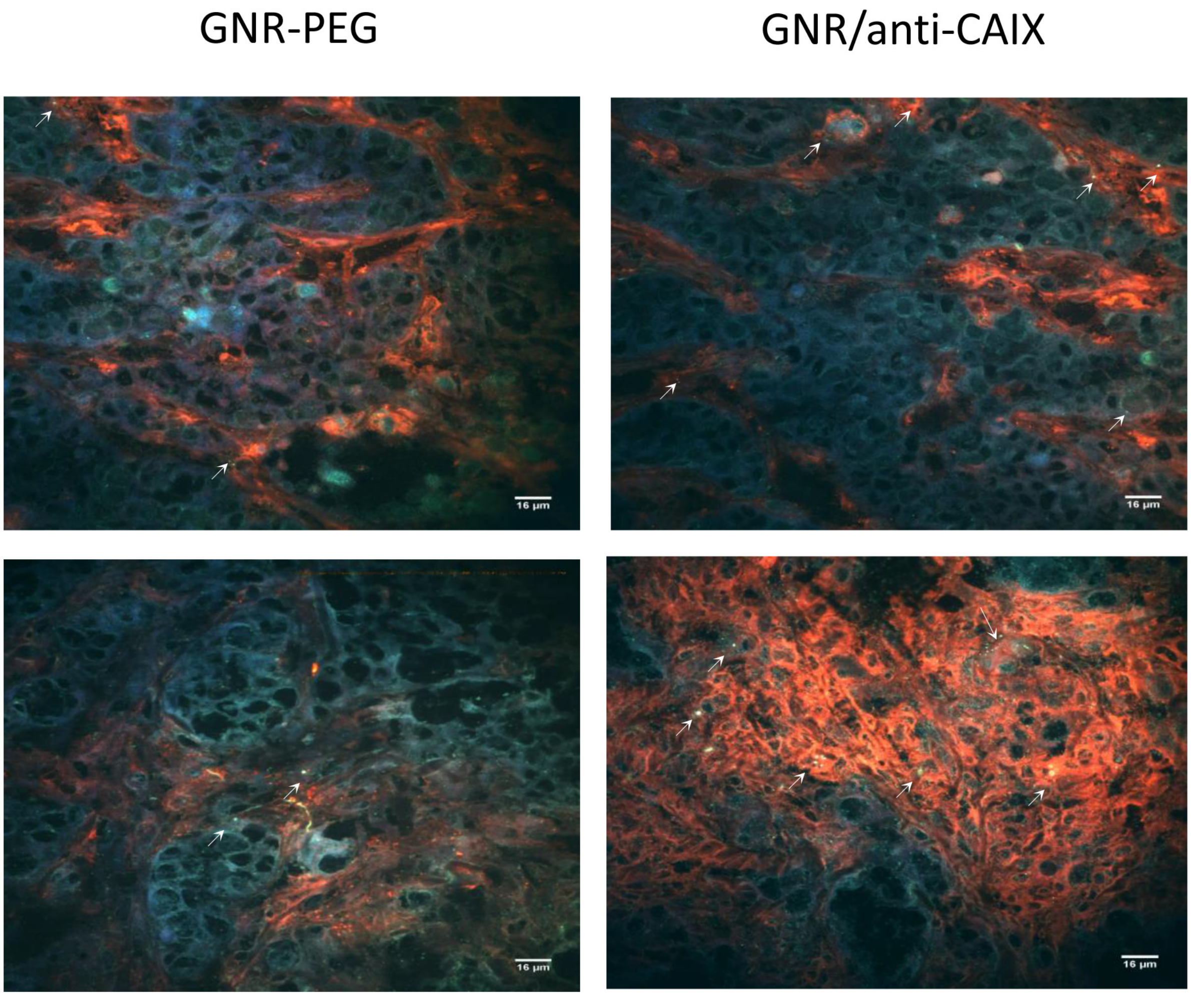

Supplementary Figure 5: Additional 60x hyperspectral immunofluorescence (orange) images of tumor hypoxia characterized by pimonidazole uptake. The GNRs in tissues (depicted with arrows) were identified by their distinct spectral characteristics.
